# Computational Systems Biology

**DOI:** 10.1155/2013/350358

**Published:** 2013-03-12

**Authors:** Xing-Ming Zhao, Weidong Tian, Rui Jiang, Jun Wan

**Affiliations:** ^1^School of Electronics and Information Engineering, Tongji University, Shanghai 201804, China; ^2^Institute of Biostatistics, Fudan University, Shanghai 200433, China; ^3^Department of Automation, Tsinghua University, Beijing 100084, China; ^4^Wilmer Eye Institute, Johns Hopkins University School of Medicine, Baltimore, MD 21287, USA

The complex biological systems consist of distinct molecules that exert their functions by interacting with each other, which makes it a big challenge to understand how the cellular machinery works. Recently, the accumulation of a large amount of multiscale omics data, such as next-generation sequencing data and protein interaction data, provides opportunity to investigate the functions of molecules from a systematic perspective. On the other hand, the analysis of these huge datasets demands efficient and robust computational methods. In this special issue, we reported the recent progress made in developing new computational methodologies to analyze the genomics data, construct gene networks, and identify disease genes. 

*Understanding the Functions of Molecules in the Postgenomic Age*. In recent years, the advance of next-generation sequencing (NGS) technology makes it more easier for researchers to access and analyze genetics data and has influential effects on the biomedical research community. However, compared with sequencing, computational analysis of the flooding sequencing data with appropriate tools is becoming a more important task when interpreting the data. In their review paper, M. P. Dolled-Filhart et al. described the pipeline for bioinformatics analysis of the NGS data, starting from alignment to variant calling as well as filtering and annotation. In each step, they discussed the tools or software that should be used as well as their advantages and caveats. This survey of the bioinformatics analysis of NGS data can help researchers to choose appropriate tools when dealing with the sequencing data.

Along with the sequencing technology, lines of evidence show that a lot of noncoding RNAs (ncRNAs) play important roles in various biological processes. Unlike the protein-coding genes that are well studied, the functions of most ncRNAs are not clear. Therefore, it is highly desirable to develop computational methods to predict the functions of the ncRNAs. H. Ma et al. conducted a survey about the computational approaches developed to predict and annotate the long noncoding RNAs (lncRNAs), which can help researchers to learn the progress in this filed and future directions in which bioinformaticists should work while annotating lncRNAs.

While annotating the functions of molecules, standard and controlled vocabularies are required. Hence, the ontologies that are represented as abstract description systems of knowledge are becoming more and more popular recently. At the same time, it is becoming a difficult task to calculate the semantic similarity between ontology terms quantitatively. M. Gan et al. introduced popular methods in quantitating the semantic similarity between ontology terms and their software implementations. Furthermore, they classified these methods into distinct categories and discussed their advantages and shortcomings, which can help researchers to select appropriate tools and methods when working on ontologies. 

Gene expression profiles can describe the molecular mechanisms that underlie certain phenotypes. However, while analyzing the gene expression data, it is inappropriate to treat genes independently considering genes interact with each other within the cell. O. Frings et al. proposed a network-based approach to analyze the gene expression data and applied it to investigate the development of sex-specific chicken gonad and brain tissues. By combining the chicken network and the gene expression data, they identified some sex-biased characteristics, for example, same sex-biased genes tend to be tightly connected in the network, and provided new insights into the molecular underpinnings of sex-biased genes.

*Construction and Analysis of Gene Networks*. Construction of gene regulatory networks (GRNs) is a crucial step in systems biology, where gene expression data is widely explored to infer the GRNs. However, the high dimensionality and notorious noise of the gene expression data makes it a nontrivial task to infer the GRNs. N. You et al. presented a new Laplace error penalty (LEP) model to calculate the partial correlation coefficients between genes and construct the GRNs. Compared with the popular least absolute shrinkage and selection operator (LASSO) and smoothly clipped absolute deviation (SCAD) approaches, the LEP method reached the highest precision. Except for gene expression data, integration of different data sources may improve the accuracy of inferred GRNs. H. Chen et al. surveyed the strategies to integrate distinct data sources and their effectiveness and recommended how to choose an appropriate strategy while integrating distinct data sources. N. Nakajima et al. proposed a novel network completion approach, DPLSQ, to infer gene networks. Benchmarking on artificial datasets, their proposed DPLSQ outperforms popular ARACNE and GeneNet with the highest accuracy. By investigating a 2-gene network, A. V. Spirov et al. found that gene cooption can affect the robustness of GRNs, and the findings provide new insights into the evolvability and robustness of GRNs.

Network modules are found to be functional blocks of gene networks, the identification of which is becoming a hot research topic. By taking the hierarchical modular structure into account, S. Zhang presented a new stochastic block model to detect the hierarchical modules. Applied to the real yeast gene coexpression network, the proposed method can efficiently detect the hierarchical modular structures that are consistent with biological functions. Recently, it is found that a particular type of ncRNAs, microRNAs, plays important roles in gene regulation by working together with transcription factors. W. Mu et al. proposed a new local genetic algorithm to predict condition-specific regulatory modules that consist of microRNAs, transcription factors, and their commonly regulated genes, and these modules provide useful insights into the regulatory mechanisms underlying gene expression. 

*Computational Approaches to Hunting Disease-Associated Genes*. The identification of genetic variants that are responsible for human diseases is critical for understanding the development of diseases and designing new effective drugs. Thanks to the genome-wide association studies (GWASs), some genetic variants that drive diseases have been identified, among which single nucleotide polymorphisms (SNPs) and nonsynonymous single nucleotide polymorphisms (nsSNPs) are receiving more and more attention. In this issue, J. Wu and R. Jiang reviewed the databases that collect nsSNPs and summarized popular computational methods that identify deleterious nsSNPs. In addition, they introduced machine learning models that are useful in predicting deleterious nsSNPs. Beyond SNP-based association analysis, gene-based association analysis is receiving increasing attention. X. Guo et al. comprehensively compared these two approaches on the data from the study of addiction and found that these two approaches complement with each other and can get better results when used together.

The differentially expressed genes identified from microarray data are generally regarded as candidate disease genes. However, the number of differentially expressed genes may reach hundreds or even thousands, thereby making it difficult to identify the potential disease genes. In this issue, L. Li et al. proposed a new hybrid approach to predict disease genes based on estimation of distribution algorithm and support vector machine. Benchmarking on B-cell lymphoma and colon cancer datasets, their method outperforms two other popular approaches and identify some new candidate genes for future validation.

